# Investigation of Bulk Traps by Conductance Method in the Deep Depletion Region of the Al_2_O_3_/GaN MOS Device

**DOI:** 10.1186/s11671-017-2111-z

**Published:** 2017-05-10

**Authors:** Yuanyuan Shi, Qi Zhou, Anbang Zhang, Liyang Zhu, Yu Shi, Wanjun Chen, Zhaoji Li, Bo Zhang

**Affiliations:** State Key Laboratory of Electronic Thin Films and Integrated Devices, University of Electronic Science and Technology of China, Chengdu, Sichuan 610054 China

**Keywords:** Al_2_O_3_/GaN MOS channel device, Conductance method, Buffer traps, Interface traps

## Abstract

Conductance method was employed to study the physics of traps (e.g., interface and bulk traps) in the Al_2_O_3_/GaN MOS devices. By featuring only one single peak in the parallel conductance (*G*_p_/ω) characteristics in the deep depletion region, one single-level bulk trap (*E*_C_-0.53 eV) uniformly distributed in GaN buffer was identified. While in the subthreshold region, the interface traps with continuous energy of *E*_C_-0.4~0.57 eV and density of 0.6~1.6 × 10^12^ cm^−2^ were extracted from the commonly observed multiple *G*_p_/ω peaks. This methodology can be used to investigate the traps in GaN buffer and facilitates making the distinction between bulk and interface traps.

## Background

Owing to the superior properties of high electron mobility, high breakdown voltage, high-power density, low on-resistance, and high temperature operation capability, GaN heterojunction field-effect transistors (HFETs) have been considered as a promising solution for next-generation energy-efficient power electronics and attracted tremendous attention in the last two decades [1]. For power switching applications, the enhancement-mode (E-mode) transistors are highly preferred rather than the depletion-mode (D-mode) devices for the inherent fail-safe operation and simple gate driver circuitry. Despite the various technologies proposed to realize E-mode, GaN HFETs such as p-cap gate [2, 3], fluorine plasma ion implantation [4], and cascode technology [5], the MOSFET with partially or fully recessed gate is considered as a promising candidate because of its high-threshold voltage (*V*_TH_), large gate swing for improved fail-safe capability [6, 7], and low on-resistance [8]. Moreover, the MOS-gate is compatible with the mainstream gate driver ICs. However, the traps (e.g., interface and bulk traps) tarnish the advantages of GaN HFETs due to the stability and reliability issues such as *V*_TH_ instability [9], drain lag or gate lag [10], and power slump. Besides the surface/interface traps, the GaN power HFETs’ performance such as the breakdown voltage and dynamic on-resistance could be substantially affected by the bulk traps in GaN buffer layer in high-voltage-switching applications [11, 12] since the high electric-field is prone to trigger the buffer traps for dynamic charging/discharging. Hence, it is of great significance to characterize the buffer traps of GaN MOS devices.

Bulk traps in GaN MOS devices have been studied by deep-level transient spectroscopy (DLTS) [13] and pulse measurement [14]. However, though the dynamic charge/discharge process of both bulk and interface trap-induced transient behavior may simultaneously appear in the same spectrum, extra effort is required to differentiate between the bulk and interface traps when using DLTS-like techniques and pulse measurement [15, 16]. On the other hand, the conductance method has been widely used to evaluate the interface traps in AlGaN/GaN MIS structures as well as GaN-based MIS structures [17–19]. Moreover, it is possible to discriminate the bulk and interface traps in the conductance method by studying its bias dependence because the conductance loss is sensitive to the traps within a few kT/q around the Fermi level. In this letter, the conventional conductance method normally used to characterize the interface traps is employed to study the bulk traps (BT) in GaN buffer for the first time. Two trap-dominated regions were found in the Al_2_O_3_/GaN MOS structure with full barrier recess. In the deep depletion region, only one single *Gp*/ω peak is captured at the measured bias voltage ranging from −1 to 0 V, revealing the bulk traps with a single level in GaN buffer. The energy of the bulk trap was determined to be *E*_C_-0.53 eV. While in the subthreshold region, the interface traps with continuous energy levels that result in multiple *Gp*/ω peaks were observed within the measured bias range of 1.6~2.6 V. The energy levels were extracted to be in the range of 0.4 to 0.57 eV below GaN conduction band (CB).

## Methods

**Fig. 1:**
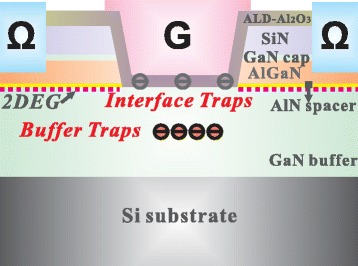
The schematic cross section of the Al_2_O_3_/GaN MOS device with full AlGaN-barrier removal

The device structure used in this work is shown in Fig. 1. The Al_2_O_3_/GaN MOS device is fabricated on a commercial Al_0.25_Ga_0.75_N/GaN heterostructure grown on a 4-in Si (111) substrate by MOCVD. The AlGaN layer was fully recessed by using the low-damage hybrid recess technique. The detail fabrication process can be found in our previous work [7]. A 20-nm Al_2_O_3_ layer was deposited by atomic layer deposition (ALD) as the gate oxide. The conductance-frequency (*G*-*f*) and capacitance-voltage (*C-V*) characteristics were measured by an Agilent B1500A Semiconductor Device Analyzer equipped with a Cascade probe station. The frequency-dependent conductance measurements are shown within the range of 1 to 5 MHz.

## Results and Discussion

**Fig. 2:**
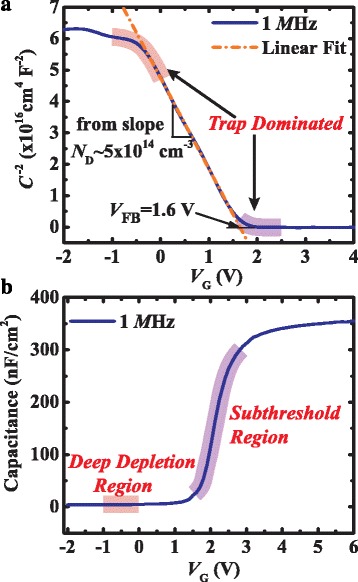
**a** 1/*C* ^2^ *-V*_G_ characteristic on Al_2_O_3_/GaN MOS device measured at frequency of 1 MHz. The *dashed dot line* is the linear fitting of the linear part of the 1/*C* ^2^ *-V*_G_ curve and the deviation from the linear slope as highlighted in the curves suggesting trap-dominated regions. **b** Two trap-dominated regions highlighted in the *C-V*_G_ curve and marked with deep depletion and subthreshold region

The 1/*C* ^2^ *-V*_G_ and *C-V*_G_ characteristics of the Al_2_O_3_/GaN MOS device measured at frequency of 1 MHz are shown in Fig. 2. A linear region was observed in the 1/*C* ^2^ *-V*_G_ curve in the gate bias voltage range of 0–1.5 V in Fig. 2a. The flat band voltage (*V*_FB_) is determined to be 1.6 V by linear extrapolation of the 1/*C* ^2^ *-V*_G_ curves to the intercept with the abscissa [20]. The background-doping concentration of GaN buffer *N*_*D*_ was extracted to be 5 × 10^14^ cm^3^ from the linear slope. The good linear fitting suggests negligible trap states in the depletion region, since the linearity of the slope is strongly affected by the charge/discharge process of the traps [21, 22]. However, a decrease in slope implies the existence of residual trap states with the applied gate voltage both lower than 0 V and higher than 1.5 V, which corresponds to the deep depletion region and subthreshold region as highlighted in Fig. 2b.

In order to further differentiate the types of the traps (e.g., interface trap or bulk trap) and study the trap characteristics including the trap levels and densities, *f*-dependent conductance (*Gp*/ω) measurements were performed. The *Gp*/ω as a function of radial frequency (*ω* = 2π*f*) can be correlated to the traps density *D*_T_ and trap response time *τ*_T_. For the case of bulk trap states with discrete energy level, *Gp*/ω is given by [23, 24]

1 $$ \frac{G_p}{\omega}=\frac{q\omega {\tau}_T{D}_T}{1+{\omega}_T^2{\tau}_T^2}, $$

whereas, for interface trap states with distributed energy levels, *Gp*/ω is given by

2 $$ \frac{G_p}{\omega}=\frac{q{ D}_T}{2\omega {\tau}_T} \ln \left(1+{\omega}_T^2{\tau}_T^2\right). $$

**Fig. 3:**
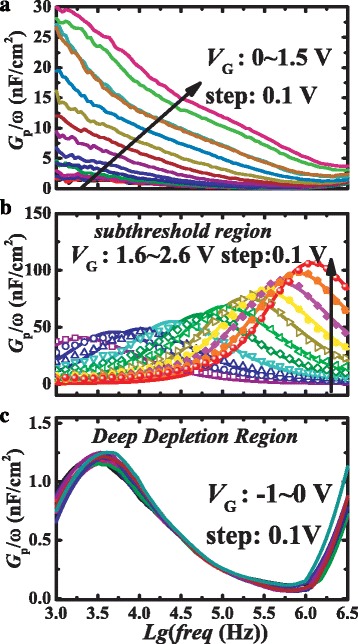
Typical frequency-dependent parallel conductance (**a**) with 0 < *V*_G_ < 1.5 V (**b**) with 1.5 V < *V*_G_ < 2.6 V (subthreshold region) (**c**) with *V*_G_ < 0 V (deep depletion region); the *dots* are the experimental data and the *solid lines* are fitting curves

The maximum loss can be obtained when the trap states are in resonance with the applied *AC* signal for dynamic discharging/charging, which occurs when the trap states are exactly half-filled, i.e., when the energy level of the trap states crosses with the semiconductor Fermi level [24]. Consequently, the trap time constant corresponds to the maximum of *Gp*/*ω* and can be determined by setting the derivative ∂(*Gp/ω*)/∂(ωτ_T_) to zero. By following the above two equations, the *ωτ*_*T*_ is found to be 1 and 1.98 for bulk traps and interface traps, respectively. Thus, the *Gp*/ω peak frequency is associated with the trap energy level and the peak value is related to the trap density. The *Gp*/ω curves monotonically decrease with the increasing frequency without *Gp*/ω peaks at 0 V < V_G_ < 1.5 V (see Fig. 3a) that corresponds to the observed linear regime shown in Fig. 2a, which reinforces the negligible charge/discharge processes of traps in this region. On the other hand, as shown in Fig. 3b, multiple peaks were observed in the *Gp*/ω curves while the device operated in the subthreshold region as the applied bias above the flat band voltage. In this region, the peaks steadily shift to higher frequencies with the increasing applied voltage, which is the typical *Gp*/ω characteristic indicating the presence of interface traps with continuous energy levels as commonly observed in conventional conductance measurements [17–19, 23]. The *Gp*/ω characteristics in the deep depletion region (−1 V < *V*_G_ < 0 V) are plotted in Fig. 3c which exhibits quite a difference compared with that observed in the subthreshold region. The *Gp*/ω curves within the measured bias range featured an identical profile with a single peak and the same *Gp*/ω peak value, which suggests the existence of trap state with only one single energy level while the electron emission rate of the trap state is irrelevant with *V*_G_. It is well known that bulk traps stem from the defects, and impurities are usually in a uniform-distribution throughout the bulky semiconductor. Correspondingly, the bulk traps are in discrete energy levels and capable of inducing the same loss peaks at the same frequency under various biases [24]. Hence, the *Gp*/ω characteristic with one peak observed in the deep depletion region reinforced that the bulk trap with one single level in GaN buffer layer was identified by conventional conductance measurement.

**Fig. 4:**
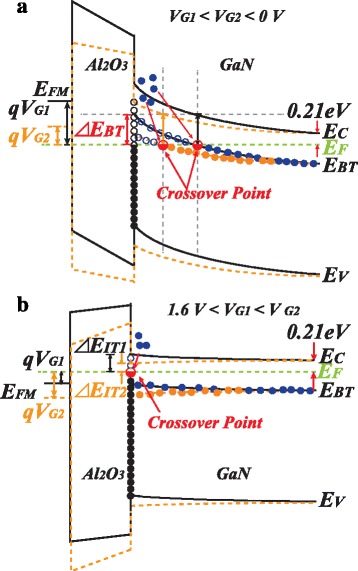
**a** The energy band diagram in depletion region to illustrate the conductance maximum loss induced by buffer traps irrelevant with *V*_G_. **b** Energy band bending in the subthreshold region to illustrate the conductance maximum loss induced by interface traps correlated with *V*_G_

The two types of traps (e.g., bulk and interface traps) can be easily discriminated by studying the bias-dependent conductance in different operation regions of the device as illustrated in the energy bandgap diagram of Al_2_O_3_/GaN MOS device in Fig. 4. The conductance method is sensitive to traps within a few kT/q around the Fermi level marked as the crossover point in Fig. 4. As shown in Fig. 4a, the single-level bulk traps are uniformly distributed in GaN buffer. The magnitude of *Gp*/ω peak originates from the bulk traps not varying with gate bias regardless of its spatial location. Meanwhile, the interface traps that cross over the Fermi level are much deeper in the GaN bandgap than the bulk trap at the given bias voltage. Therefore, the conductance loss was dominated by the dynamic response of the bulk traps rather than the interface traps in the deep depletion region. Further sweep up the bias voltage (e.g., 0 V < *V*_G_ < 1.5 V), all of the bulk traps were filled with electrons as shown in Fig. 4b. On the other hand, the interface traps with a wide energy distribution may still capture the electrons. However, the conductance loss was not detected at the measured frequencies (e.g., 1 kHz–5 MHz) due to the extremely large time constant associated with the deep levels of the interface traps. Consequently, the 1/*C* ^2^ *-V*_G_ plot exhibits a linear characteristic and the *Gp*/ω curves show a monotone property while 0 V < *V*_G_ < 1.5 V as shown in Fig. 2a and Fig. 3a, respectively. In the subthreshold region (1.6 V < *V*_G_ < 2.6 V), it can be seen from Fig. 4b that the conduction loss is solely contributed by the charging/discharging of the interface traps. Because the energy level of the interface traps that cross over the Fermi level are shallower in the subthreshold region, the relatively small time constant for dynamic charging/discharging can be detected as the multiple *Gp*/ω peaks measured in Fig. 3b.

**Fig. 5:**
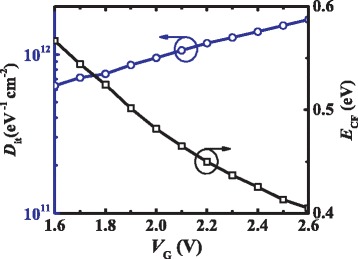
Plots of *D*_IT_ and the interface trap energy level relative to the *CB* (∆*E*_IT_ *= E*_C_-*E*_IT_) for each applied gate voltage in the subthreshold region

By using Eq. (2), the fitting curves exhibit good agreement with the measured *Gp*/ω characteristics in the subthreshold region. The interface trap density and energy distribution profile were extracted and plotted versus the applied voltage in Fig. 5. The trap energy levels relative to the CB *∆E*_T_ as function of response time *τ*_*T*_ given by the Shockley–Read–Hall statistics were extracted using the equation [14]:

3 $$ \varDelta {E}_T={k}_B T \ln \left({v}_{th}{\sigma}_n{N}_C{\tau}_T\right), $$

where the capture cross section of the trap *σ*_n_ = 4 × 10^−13^ cm^−2^, the electron thermal velocity *v*_th_ =2.6 × 10^7^ cm/s, the density of states at GaN CB *N*_C_ = 2.2 × 10^18^ cm^−3^, the Boltzmann constant *k*_B_ = 1.38 × 10^−23^J/K, and temperature *T* = 300 K were used [25]. The interface trap levels are in the range of 0.4 to 0.57 eV below GaN CB with *D*_IT_ decreased from 1.6 × 10^12^ to 0.6 × 10^12^cm^−2^.

**Fig. 6:**
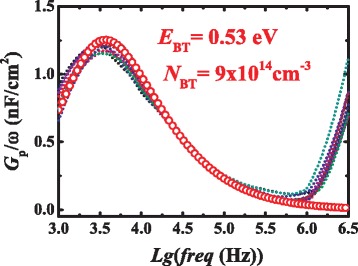
Experimental (*dotted lines*) and fitted (*red circles*) *Gp*/ω curves with gate biased in the deep depletion region

Similarly, the energy level and density of bu traps in GaN buffer also can be extracted by fitting the *Gp*/ω characteristics with Eqs. (1) and (3) as shown in Fig. 6. More importantly, as the measured *Gp*/ω characteristics exhibit an identical profile with only one single peak value at various bias voltages, the measured data were well fitted by a single curve instead of a series *Gp*/ω curves for the interface trap that feature a continuum energy-level distribution. Thus, single-level bulk traps with sheet density 1.5 × 10^10^ cm^−2^ and *E*_BT_ = *E*_C_-0.53 eV (close to the reported Fe-induced level at 0.5 ± 0.1 eV below the GaN conduction band edge in GaN buffer [26, 27]) was extracted. Accordingly, the volume density *N*_BT_ of the buffer trap can be obtained by dividing the sheet density by Debye length ${L}_D=\sqrt{kT{\varepsilon}_S{\varepsilon}_0/\left({q}^2{N}_D\right)}$ as the depletion depth being in the same order of magnitude or smaller than the Debye length [28]. With the unintentional doping density *N*_*D*_ = 5 × 10^14^cm^−3^ extracted from the slope of the 1/*C* ^2^ *-V*_*G*_ curve, *N*_BT_ was estimated to be 9 × 10^14^cm^−3^.

## Conclusions

In conclusion, for the first time, the conventional conductance method was used to study the buffer traps in the Al_2_O_3_/GaN MOS device with full barrier removal. The bulk traps with a single energy level and uniformly distributed in GaN buffer that leads to a single *Gp*/ω peak were detected by *f*-dependent conductance measurements in the deep depletion region. On the other hand, the interface traps with wide energies were measured in the subthreshold region, which corresponds to the multiple *Gp*/ω peaks observed in the *f*-dependent conductance measurements. Due to the different *f-*dependent conductance response originating from the different energy and spatial distributions, the demonstrated approach is much easier to be used to investigate the physics of the bulk traps in GaN buffer.
